# Minimal effective dose of lactic-fermented egg white on visceral fat in Japanese men: a double-blind parallel-armed pilot study

**DOI:** 10.1186/s12944-019-1047-y

**Published:** 2019-04-22

**Authors:** Ryosuke Matsuoka, Keiko Kamachi, Mika Usuda, Yasunobu Masuda, Masaaki Kunou, Akira Tanaka, Kazunori Utsunomiya

**Affiliations:** 1 R&D Division, Kewpie Corporation, 2-5-7 Sengawa Kewport, Sengawa-Cho, Chofu-Shi, Tokyo, 182-0002 Japan; 2 Nutrition Clinic, Kagawa Nutrition University, Komagome, Toshima-ku, Tokyo, 170-8481 Japan; 3 Division of Diabetes, Metabolism & Endocrinology, Jikei University School of Medicine, 3-25-8, Nishi-shinbashi, Minato-ku, Tokyo, 105-8461 Japan

**Keywords:** Egg white, Visceral fat, Obesity, Japanese, Protein

## Abstract

**Background:**

We previously reported that the consumption of 8 g of protein per day in the form of lactic-fermented egg white (LAFEW) improves visceral fat obesity. In this study, we investigated the minimum effective intake of LAFEW for visceral fat reduction in the Japanese males with mild obesity.

**Methods:**

Twenty-two Japanese adult males with a Body mass index (BMI) ≥24 and a waist circumference ≥ 85 cm were included in this study. The subjects were divided into three groups, that is, control group, LAFEW 6 g group, and LAFEW 8 g group. The LAFEW 6 and 8 g groups consumed 6 and 8 g, respectively, of egg white protein (EWP) in a drink at breakfast for 8 weeks, whereas the control group consumed a drink containing 8 g of milk whey protein. Body weight, body fat percentage, abdominal circumference, and visceral fat (VF) area around the navel were measured at 0 and 8 weeks after initiating the consumption.

**Results:**

No changes in body weight or body fat percentage were observed in any of the groups. No significant differences between the pre- and posttreatment measurements were found in the VF area around the navel in the control group and the LAFEW 6 g group. In the LAFEW 8 g group, the VF area had decreased significantly after 8 weeks of consumption, when compared to that before consumption, and the average observed decrease (Δcm^2^) was 13.2 ± 4.7 cm^2^. Among the subjects with an initial BMI > 25, the VF area was significantly smaller in the LAFEW 8 group, when compared to the week 0 values and those in the control group. Visceral fat/subcutaneous fat values in the LAFEW 8 group were also significantly smaller than those in the control group or at week 0.

**Conclusion:**

The results suggested that the minimum effective intake of EWP in the LAFEW to reduce the VF area in the Japanese men is 8 g.

**Trial registration:**

This clinical trial was retrospectively registered with the University hospital Medical Information Network (UMIN) Center, (UMIN000031681; registered on 12/03/2018).

## Introduction

Egg white protein (EWP) has an amino acid score of 100 [1, 2], and has been reported to retain a high level of bioavailability even when not heated, or after half-boiling or heating cooking processes [3]. We have previously reported, in an animal experiment, that the consumption of egg white protein increases the body’s protein mass while decreasing the body fat and visceral fat [4].

However, egg white is difficult to intake as it is because of its flavor and physical properties. Therefore, we subjected the egg whites to lactic fermentation to develop a LAFEW that is easier to drink [5].

We previously reported that a daily intake of 8 g EWP by LAFEW for 12 weeks reduces the amount of visceral and visceral/subcutaneous fats and significantly relieves visceral fat obesity when compared to the preconsumption levels or control whey protein treatment [6]. The effects of less than 8 g daily intake of EWP on the reduction of visceral and visceral/subcutaneous fats remain unclear.

Similar to the egg white protein, lactoferrin and soybean-derived β-conglycinin have been reported to reduce visceral fat at daily intakes of 300 mg and 5 g, respectively [7, 8]. The inhibition of lipid absorption has been reported as a mechanism for the reduction of visceral fat by β-conglycinin [9], whereas the inhibition of lipid absorption by ovalbumin, ovotransferrin, and lysozyme has been reported as a mechanism underlying the visceral fat reduction effect of egg white protein [10, 11]. To ingest 5 g as these 3 protein fractions, 8 g daily doses of EWP must be ingested [6, 12].

EWP is known to contain ovotransferrin, which has a similar function to that of lactoferrin. If ovotransferrin has an equivalent physiological activity as lactoferrin, an estimated daily intake of 3 g EWP should be sufficient to reduce visceral fat with LAFEW. Therefore, the minimum effective dose of EWP was hypothesized to be 3–8 g per day.

In a study investigating the effects of LAFEW on serum cholesterol levels, an 8-week daily consumption of 8 g EWP reduced the total and LDL cholesterol in serum to levels significantly lower than those before intake (− 4.7, − 9.2%, respectively) or after consumption of the 4 g daily intake group [13]. After 8 weeks of daily consumption of 6 g of EWP, serum LDL-cholesterol concentrations were also significantly lower than those before consumption (− 2.5, − 6.4%, respectively) [13]. No changes were observed in the total and LDL cholesterol levels in serum when a daily dose of 4 g of EWP was used (+ 1.6%, − 1.3%, respectively) [13].

Therefore, the 8-week consumption of EWP at a daily intake of 6 g was anticipated to reduce visceral fat as it had previously improved lipid metabolism.

In this study, we evaluated the minimum effective daily intake dose for visceral fat reduction in humans using LAFEW preparations containing 6 and 8 g of EWP.

## Methods

### Test food

The control whey protein drink and the drink containing LAFEW were prepared at R&D Division, Kewpie Corporation., Tokyo. Each drink was prepared by adding flavor, sweetener, water, etc., to milk whey (Nippon Shinyaku Co., Ltd., Kyoto) or LAFEW [5] (Kewpie Egg Corporation, Tokyo), by uniform mixing of the solution and heat-sterilizing. Nutritional value of whey drink and LAFEW drink were same (Energy 64 and 60 kcal/100 g, protein 4.4 and 4.5 g/100 g, Lipids 0.4 and 0.1 g/100 g, Carbohydrate 10.8 or 10.5 g/100 g, respectively). The protein content of LAFEW or whey drink was determined using the Kjeldahl method [14], and we measured 8 g protein (EWP)/180 g in the control drink and LAFEW 8 g drink and as 6 g protein (EWP)/135 g for the LAFEW 6 g drink. Ovalbumin content in LAFEW was assessed by the sandwich ELISA procedure using an anti-chicken ovalbumin polyclonal antibody, followed with a horseradish peroxidase-labeled anti-chicken ovalbumin polyclonal antibody. We used the commercial Egg (Ovalbumin) ELISA kit II (Morinaga Institute of Biological Science Inc., Yokohama, Japan) at a detection absorbance of 450 nm using a multidetection microplate reader (Powerscan® HT, DS Pharma Biomedical Co. Ltd., Osaka, Japan). Observed ovalbumin content in our LAFEW drink using the sandwich ELISA was 3.92 g/180 g.

### Subjects and test methods

Twenty-two adult males with a BMI > 24 and a waist circumference of > 85 cm served as subjects in this study. The subjects did not undergo treatment for hyperlipidemia or diabetes, had no subjective symptoms of gout, were capable of visiting the designated institution as scheduled, and were able to fill the forms required for the study, such as self-diagnosis forms. The exclusion criteria were as follows: intake of medications that could potentially affect the test results (e.g., antihyperlipidemic agents, antidiabetic agents, oral corticosteroid formulations, or antihypertensive agents); regular consumption of foods for specified health uses that could potentially affect the test results; excessive alcohol consumption; suspected allergic reactions (particularly to egg and milk); participation in other clinical studies; a history of serious liver damage, kidney damage, or myocardial infarction; a history of, or present condition of, hepatitis; and serious anemia.

The subjects were divided into three groups: the control group, the LAFEW 6 g group, and the LAFEW 8 g group. The subjects in the control group, LAFEW 6 g group, and LAFEW 8 g group consumed a milk whey drink for breakfast for 8 weeks (8 g of whey protein), LAFEW with 6 g of EWP, or LAFEW with 8 g of EWP, respectively.

Abdominal CT scans (SOMATOM Emotion™, Siemens Healthcare K.K., Tokyo, Japan) were performed at weeks 0 and 12 of the study to measure the visceral fat deposits around the navel (Fat Scan, East Japan Institute of Technology Co., Ltd., Ibaraki, Japan). CT scans were performed at Kobayashi Hospital (Tokyo). At weeks 0 and 8, the subjects underwent fasting for at least 10 h before the blood samples were collected from a forearm vein and measurements were taken for body weight, body fat percentage, blood pressure, and abdominal circumference. A meal survey was conducted 3 days before blood sampling at weeks 0 and 8 to confirm that the nutritional intake remained unchanged.

### Blood analysis

General peripheral blood tests were performed using flow cytometry. Sera were used to measure the following components: total cholesterol (enzymatic method), HDL-cholesterol (direct method), triglycerides (enzymatic method), free fatty acids (enzymatic method), phospholipids (enzymatic method), glucose (hexokinase UV method), HbA1c (latex agglutination method), RLP-cholesterol (immunosorbent method), insulin (CLEIA method), AST (JSCC standardization compatible method), ALT (JSCC standardization compatible method), γ-GTP (JSCC standardization compatible method), urea nitrogen (urease·LED·UV method), creatinine (enzymatic method), and uric acid (enzymatic method). LDL-cholesterol was calculated using Friedwald’s equation [15]. Blood tests were conducted at SRL Inc. (Tokyo).

### Dietary analysis

Our meal analysis comprised subjects reporting the contents of their meals for 3 days to calculate nutrition. Excel Eiyoukun ver. 5 (Kenpakusha, Tokyo) was used to perform the nutrition calculations. The 2010 Standard Tables of Food Composition in Japan was used as a database of the nutrition calculation software.

### Statistical analysis

The test results were expressed in the form of a mean ± standard error. The paired t-test and Dunnett’s test were used to compare the treatment measurements with control and pretreatment measurements. Differences with a hazard ratio of less than 5% were considered to be statistically significant. Statistical analyses were performed using SPSS ver. 20 (SPSS Co., Ltd.).

## Results

### Subject background

**Fig. 1:**
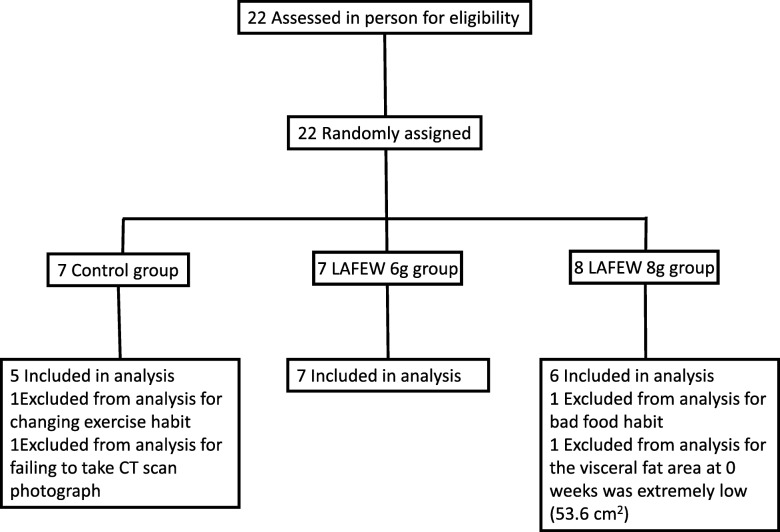
Participant flow through a randomized trial

No significant differences in age, body height, body weight, BMI, and systolic and diastolic blood pressure values were found among the three groups of subjects, confirming adequate group distribution (Table 1). All subjects successfully completed the study, with no withdrawals; however, certain inconsistencies were observed among the subjects; in the control group, one subject exercised excessively and one subject’s CT imaging resulted in an error, and in the LAFEW 8 g group, one subject had low VF area (53.6 cm^2^) at the commencement of the study and one subject was prescribed a cholesterol-lowering drug by a physician during the study period. Therefore, these subjects were excluded from the analyses. As a result, analyses included 5 subjects in the control group, 7 subjects in the LAFEW 6 g group, and 6 subjects in the LAFEW 8 g group (Fig. 1).

**Table 1:** Background of Subjects

	Control	LAFEW 6 g	LAFEW 8 g
Age (y)	53.2 ± 3.1	54.9 ± 2.2	52.2 ± 3.2
Height (cm)	174 ± 2	171 ± 1	172 ± 2
Body weight (kg)	80.3 ± 2.8	76.6 ± 2.5	83.6 ± 3.7
BMI	26.6 ± 0.9	26.3 ± 0.8	28.5 ± 1.5
Systolic blood presure (mmHg)	142 ± 6	147 ± 6	146 ± 10
Diastolic blood pressure (mmHg)	86.6 ± 3.9	94.0± 3.0	90.2 ± 6.4

Mean ± SE of 5 (Control), 7 (LAFEW 6 g), or 6 (LAFEW 8 g)

Control: Control group, LAFEW 6 g: LAFEW 6 g group, LAFEW 8 g: LAFEW 8 g group

*LAFEW* Lactic-fermented egg white

### Dietary analysis

Significant changes in nutrition intake were observed over the course of the study period. In the LAFEW 6 g group, the carbohydrate intake decreased significantly from the preconsumption values; however, no significant changes in the energy intake were noted between the pre- and postconsumption levels. In the control group, salt intake increased significantly from the preconsumption values; however, a causal relationship between salt intake and visceral fat accumulation has not been reported, and no significant increases were observed in the blood pressure values, which we presumed to have been affected by the salt intake.

In both LAFEW groups (6 and 8 g), energy and protein intake at week 8 were significantly lower than the respective values in the control group. The dietary fiber intake in the LAFEW 6 g group was significantly lower than that in the control group, and the cholesterol intake in the LAFEW 8 g group was significantly lower than that in the control group; however, no significant differences were observed in the dietary fiber and cholesterol intake in these groups between weeks 0 and 8, and no significant changes in intake were observed over the course of the study (Table 2). We calculated change % of dietary intake.

**Table 2:** Dietary Intake of Subjects

		Intake Periods (Weeks)		Change (%)
		0	8	
Energy (kcal)	Control	2114 ± 126	2303 ± 94	113 ± 5
	LAFEW 6 g	1857 ± 59	1698 ± 96#	91.6 ± 5.1
	LAFEW 8 g	2048 ± 144	1850 ± 97#	92.7 ± 7.7
Protein (g)	Control	71.6 ± 7.7	84.5 ± 2.0	127 ± 13
	LAFEW 6 g	71.3 ± 4.3	60.9 ± 4.1#	87.4 ± 8.4
	LAFEW 8 g	81.1 ± 7.1	65.0 ± 7.5#	83.5 ± 11.0
Lipids (g)	Control	71.4 ± 6.4	78.7 ± 6.6	119 ± 11
	LAFEW 6 g	56.6 ± 4.4	56.5 ± 7.0	102 ± 11
	LAFEW 8 g	71.2 ± 6.0	57.0 ± 5.8	94.7 ± 11.8
Carbohydrate (g)	Control	278 ± 15	298 ± 13	109 ± 4
	LAFEW 6 g	246 ± 19	213 ± 23*#	85.6 ± 4.3#
	LAFEW 8 g	262 ± 27	261 ± 13	104 ± 10
Cholesterol (mg)	Control	367 ± 91	439 ± 63	170 ± 52
	LAFEW 6 g	328 ± 30	324 ± 43	101 ± 12
	LAFEW 8 g	285 ± 35	235 ± 30#	93.4 ± 22
Dietary fiber (g)	Control	13.2 ± 2.1	12.7 ± 1.6	102 ± 9
	LAFEW 6 g	10.5 ± 1.1	8.97 ± 0.97#	87.1 ± 7.7
	LAFEW 8 g	12.2 ± 1.8	10.2 ± 1.1	87.7 ± 8.5

Mean ± SE of 5 (Control), 7 (LAFEW 6 g), or 6 (LAFEW 8 g)

Control: Control group, LAFEW 6 g: LAFEW 6 g group, LAFEW 8 g: LAFEW 8 g group

*p < 0.05 vs. 0 week by paired *t*-test, #*p* < 0.05 vs. Control by Dunnett test

*LAFEW* Lactic-fermented egg white

### Physical condition

For all items, no significant differences in physical condition were noted between weeks 0 and 8 or between the control group and the LAFEW 6 or 8 g groups (Table 3).

**Table 3:** Results of Body Composition

		Intake Periods (Weeks)		Change (%)
		0	8	
Body weight (kg)	Control	80.3 ± 2.8	80.7 ± 2.8	101 ± 1
	LAFEW 6 g	76.6 ± 2.5	76.8 ± 2.3	100 ± 1
	LAFEW 8 g	83.6 ± 3.7	83.2 ± 4.2	100 ± 1
BMI	Control	26.6 ± 0.9	26.7 ± 0.9	101 ± 1
	LAFEW 6 g	26.3 ± 0.8	26.3 ± 0.7	100 ± 1
	LAFEW 8 g	28.5 ± 1.5	28.4 ± 1.7	100 ± 1
Waist (cm)	Control	95.6 ± 2.1	95.2 ± 2.4	99.6 ± 0.8
	LAFEW 6 g	93.8 ± 1.5	92.8 ± 1.8	99.0 ± 0.6
	LAFEW 8 g	98.5 ± 1.7	97.0 ± 2.1	98.4 ± 0.8
Total fat area (cm^2^)	Control	315 ± 42	316 ± 44	101 ± 3
	LAFEW 6 g	310 ± 15	317 ± 16	103 ± 3
	LAFEW 8 g	365 ± 37	358 ± 38	98.0 ± 2.7
Subcutaneous fat area (cm^2^)	Control	179 ± 17	179 ± 17	100 ± 1
	LAFEW 6 g	160 ± 18	168 ± 20	104 ± 2
	LAFEW 8 g	223 ± 26	230 ± 29	103 ± 3
Visceral fat area (cm^2^)	Control	137 ± 28	137 ± 29	102 ± 8
	LAFEW 6 g	149 ± 18	149 ± 16	101 ± 4
	LAFEW 8 g	141 ± 14	128 ± 15*	89.9 ± 3.8
Visceral fat/Subcutaneous fat	Control	0.753 ± 0.115	0.743 ± 0.100	101 ± 8
	LAFEW 6 g	1.03 ± 0.19	0.994 ± 0.189	96.4 ± 1.9
	LAFEW 8 g	0.647 ± 0.051	0.577 ± 0.064*	88.2 ± 4.5

Mean±SE of 5 (Control), 7 (LAFEW 6g), or 6 (LAFEW 8g)

Control: Control group, LAFEW 6g: LAFEW 6g group, LAFEW 8g: LAFEW 8g group

*p<0.05 vs. 0weeks by paired t-test

*LAFEW* Lactic-fermented egg white

### Visceral fat area (Table 3)

No significant differences from the preconsumption values were found among the three groups while measuring the total and subcutaneous fat area. In addition, no significant differences were observed in total and VF area between the LAFEW 6 or 8 g groups and the control group (Table 3).

The LAFEW 8 g group revealed significantly reduced VF area over the course of the study, with a mean decrease of 13.2 cm^2^, whereas no significant differences from the preconsumption values were found in the control and the LAFEW 6 g groups. No significant differences in the VF area were found among the three groups. The LAFEW 8 g group revealed significantly reduced visceral/subcutaneous fat over the course of the study, whereas no significant differences were observed in the control and the LAFEW 6 g groups. In addition, no significant differences in total fat area and VF area were noted between the LAFEW 6 or 8 g groups and the control group (Table 3).

**Fig. 2:**
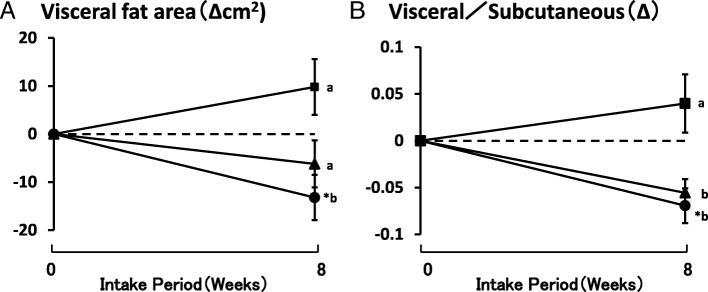
Change in VF area and ratio of visceral to subcutaneous fat area (BMI > 25). Mean ± SE of 4 (control), 4 (LAFEW 6 g), and 6 (LAFEW 8 g), **a**: results of Visceral fat area (Δcm2), **b**: Result of Visceral fat/Subcutaneous fat (Δ), ■: Control group, ▲: LAFEW 6 g group, ●: LAFEW 8 g group, *: *p* < 0.05 vs. 0 weeks by paired *t*-test, Diffecent letters shown a significant Difference by Dunnett test (*p* < 0.05)

**Fig. 3:**
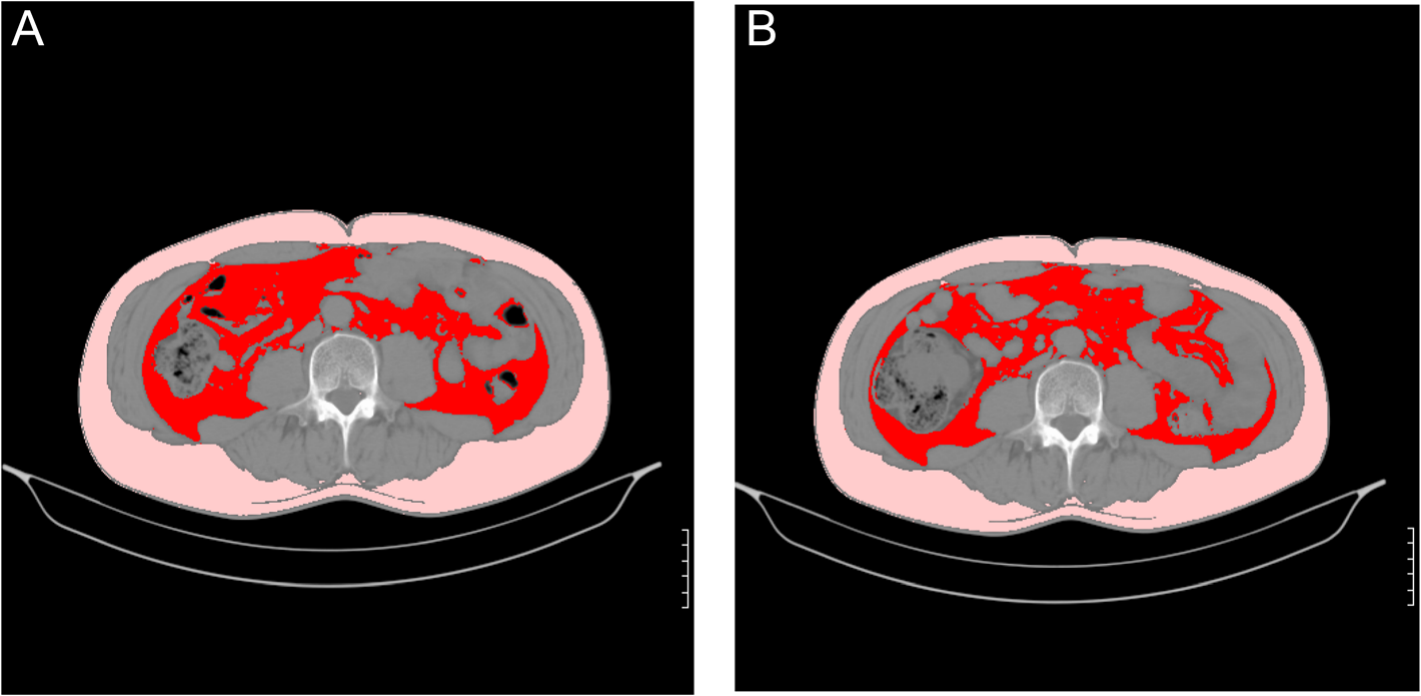
Representative CT scans demonstrating the change in visceral fat area (VFA) of the subjects fed lactic-fermented egg white (8 g as protein) for 8 weeks. Red: Visceral fat, Pink: Subcutaneous fat. **a**: Pre-intake (VF area = 107 cm^2^), **b**: Post-intake (VF area = 89 cm^2^)

The stratified analysis in the subjects with a BMI > 25 revealed that the VF area and visceral fat/subcutaneous fat in the LAFEW 8 g group were significantly reduced compared to the values in the control group or at week 0. The visceral fat/subcutaneous fat measurements in the LAFEW 6 g group were significantly lower than that in the control group, but not significantly different from the week 0 value (Figs. 2 and 3).

We excluded the patients diagnosed with obesity, impaired glucose tolerance, dyslipidemia and hypertension in this study and reanalysis was conducted to prevent the metabolic syndrome. In the LAFEW 8 g group (*n* = 3), the change in the VF area (Δcm [2]) around the umbilicus was − 18.4 ± 4.9 cm^2^, which was significantly lower than before treatment and that of the control group (*n* = 3, + 6.7 ± 6.2 cm^2^) and the LAFEW 6 g group (*n* = 4, + 5.3 ± 5.3 cm^2^).

### Hematological tests

In the control group, the mean corpuscular volume (MCV) was significantly higher and mean corpuscular hemoglobin concentration (MCHC) was significantly lower, compared to the respective values before treatment. In the LAFEW 8 g group, the red blood cell counts (RBC), hemoglobin (Hb), and MCHC values were significantly reduced and MCHC was significantly increased, compared to the pretreatment values; however, all hematological values measured remained within the respective normal ranges (Table 4). No change in the results of Hematological tests in the LAFEW 6 g group (Table 4).

**Table 4:** Results of Hematological Tests

		Intake Periods (Weeks)		Change (%)
		0	8	
WBC (/μL)	Control	6060 ± 665	5500 ± 619	91.4 ± 5.3
	LAFEW 6 g	5329 ± 467	5214 ± 528	98.1 ± 4.3
	LAFEW 8 g	6150 ± 887	5900 ± 753	97.9 ± 7.8
RBC (/μL)	Control	498 ± 16	493 ± 15	99.0 ± 1.0
	LAFEW 6 g	501 ± 20	491 ± 18	98.2 ± 1.8
	LAFEW 8 g	512 ± 7	495 ± 7*	96.6 ± 1.0
Hb (g/dL)	Control	15.2 ± 0.3	15.1 ± 0.4	99.1 ± 0.8
	LAFEW 6 g	15.4 ± 0.5	15.0 ± 0.3	97.2 ± 1.9
	LAFEW 8 g	15.7 ± 0.3	15.1 ± 0.2*	96.7 ± 1.1
Ht (%)	Control	47.4 ± 1.2	48.0 ± 1.0	101 ± 1.2
	LAFEW 6 g	47.7 ± 1.6	46.7 ± 1.1	98.1 ± 1.9
	LAFEW 8 g	48.8 ± 0.6	48.1 ± 0.5	98.7 ± 0.7
MCV (fl)	Control	95.2 ± 1.1	97.6 ± 1.5*	103 ± 0.6
	LAFEW 6 g	95.5 ± 2.3	95.3 ± 1.8	99.9 ± 0.7#
	LAFEW 8 g	95.4 ± 0.5	97.4 ± 1.1*	102 ± 0.7
MCH (pg)	Control	30.6 ± 0.6	30.6 ± 0.7	100 ± 0.5
	LAFEW 6 g	30.9 ± 0.7	30.6 ± 0.7	98.9 ± 0.6
	LAFEW 8 g	30.6 ± 0.5	30.6 ± 0.4	100 ± 0.3
MCHC (%)	Control	32.1 ± 0.3	31.4 ± 0.4*	97.6 ± 0.8
	LAFEW 6 g	32.4 ± 0.3	32.1 ± 0.2	99.1 ± 0.9
	LAFEW 8 g	32.1 ± 0.4	31.5 ± 0.3*	98.1 ± 0.8
Platelet (/μL)	Control	22.0 ± 2.6	22.1 ± 2.5	101 ± 4.0
	LAFEW 6 g	20.9 ± 2.1	21.2 ± 2.0	102 ± 5.7
	LAFEW 8 g	23.5 ± 1.4	24.9 ± 1.5	106 ± 2.6

Mean ± SE of 5 (Control), 7 (LAFEW 6 g), or 6 (LAFEW 8 g)

Control: Control group, LAFEW 6 g: LAFEW 6 g group, LAFEW 8 g: LAFEW 8 g group

*p < 0.05 vs. 0 week by paired *t*-test, #*p* < 0.05 vs. Control by Dunnett test

*LAFEW* Lactic-fermented egg white

### Serum lipid levels

No significant changes were observed in the serum levels of total cholesterol, HDL-cholesterol, LDL-cholesterol, RLP-cholesterol, triglycerides, phospholipids, and free fatty acids. In the LAFEW 6 g group, HbA1c measurements were significantly higher than that in the pretreatment levels; however, the glucose levels were also significantly lower (Table 5).

**Table 5:** Serum Biochemical Analysis

		Intake Periods (Weeks)		Change (%)
		0	8	
Total Cholesterol (mg/dL)	Control	200 ± 5	191 ± 7	95.4 ± 2.6
	LAFEW 6 g	220 ± 9	211 ± 6	96.2 ± 2.9
	LAFEW 8 g	250 ± 12#	229 ± 21	90.4 ± 5.3
HDL-Cholesterol (mg/dL)	Control	50.0 ± 4.8	51.4 ± 3.2	104 ± 5
	LAFEW 6 g	49.7 ± 3.3	49.1 ± 3.6	98.8 ± 3.6
	LAFEW 8 g	65.8 ± 5.3	56.8 ± 7.6	85.6 ± 7.9
LDL-Cholesterol (mg/dL)	Control	134 ± 5	129 ± 7	95.0 ± 3.2
	LAFEW 6 g	133 ± 8	134 ± 5	95.4 ± 5.8
	LAFEW 8 g	160 ± 12	150 ± 14	88.5 ± 5.8
Triglyceride (mg/dL)	Control	125 ± 29	103 ± 16	88.4 ± 10.3
	LAFEW 6 g	250 ± 57	230 ± 70	99.7 ± 18.1
	LAFEW 8 g	147 ± 40	166 ± 34	124 ± 13
NEFA (mEq/L)	Control	536 ± 103	351 ± 29	74.3 ± 13.5
	LAFEW 6 g	477 ± 61	498 ± 110	109 ± 19
	LAFEW 8 g	431 ± 62	540 ± 84	116 ± 15
Phospholipid (mg/dL)	Control	202 ± 8	192 ± 4	95.4 ± 3.1
	LAFEW 6 g	234 ± 12	223 ± 14	95.2 ± 2.4
	LAFEW 8 g	250 ± 14#	234 ± 22	91.9 ± 5.6
Glucose (mg/dL)	Control	98.6 ± 6.2	97.8 ± 9.9	98.5 ± 3.9
	LAFEW 6 g	99.4 ± 3.0	93.3 ± 3.1*	93.9 ± 1.9
	LAFEW 8 g	99.7 ± 5.2	95.8 ± 4.3	96.5 ± 2.6
HbA1c (%)	Control	5.44 ± 0.32	5.72 ± 0.47	104 ± 3
	LAFEW 6 g	5.13 ± 0.18	5.37 ± 0.16*	105 ± 2
	LAFEW 8 g	5.62 ± 0.20	5.65 ± 0.18	101 ± 2
RLP-Cholsterol (mg/dL)	Control	4.84 ± 0.81	3.80 ± 0.26	75.3 ± 7.5
	LAFEW 6 g	8.90 ± 1.79	8.76 ± 2.58	107 ± 22
	LAFEW 8 g	6.40 ± 1.67	5.05 ± 0.94	107 ± 11

Mean ± SE of 5 (Control), 7 (LAFEW 6 g), or 6 (LAFEW 8 g)

Control: Control group, LAFEW 6 g: LAFEW 6 g group, LAFEW 8 g: LAFEW 8 g group

*p < 0.05 vs. 0 week by paired *t*-test, #*p* < 0.05 vs. Control by Dunnett test

*LAFEW* Lactic-fermented egg white

### Hepatic and renal function indices

In the LAFEW 6 g group, creatinine measurements were significantly higher than that before consumption. The blood urea nitrogen (BUN) measurements in the LAFEW 6 g group were significantly lower than that in the control group; however, these values were within the respective normal ranges. For other items, no effects of the test foods were observed (Table 6).

**Table 6:** Results of Liver and Kidney Function

		Intake Periods (Weeks)		Change (%)
		0	8	
AST (IU/L)	Control	50.4 ± 26.7	35.2 ± 12.3	88.8 ± 9.9
	LAFEW 6 g	21.9 ± 1.9	22.1 ± 2.4	101 ± 5.4
	LAFEW 8 g	31.3 ± 6.0	28.7 ± 4.4	98.2 ± 12.2
ALT (IU/L)	Control	52.2 ± 28.2	45.4 ± 21.7	95.5 ± 6.2
	LAFEW 6 g	22.0 ± 2.8	23.4 ± 3.7	105 ± 7.2
	LAFEW 8 g	49.0 ± 12.9	39.0 ± 10.5	94.3 ± 21.4
γ-GTP (IU/L)	Control	72.6 ± 33.0	74.6 ± 35.5	105 ± 9.9
	LAFEW 6 g	70.6 ± 21.3	70.9 ± 24.4	96.7 ± 5.8
	LAFEW 8 g	37.8 ± 7.3	33.7 ± 7.3	86.8 ± 5.4
BUN (mg/dL)	Control	13.0 ± 1.3	14.2 ± 0.9	111 ± 8.2
	LAFEW 6 g	12.5 ± 1.1	11.2 ± 0.7#	92.5 ± 7.8
	LAFEW 8 g	12.8 ± 0.5	11.8 ± 0.7	91.9 ± 4.3
Cr (mg/dL)	Control	0.748 ± 0.042	0.752 ± 0.039	101 ± 2
	LAFEW 6 g	0.779 ± 0.029	0.836 ± 0.030*	108 ± 3
	LAFEW 8 g	0.798 ± 0.032	0.823 ± 0.024	104 ± 3
UA (mg/dL)	Control	6.34 ± 0.15	6.16 ± 0.37	97.7 ± 7.4
	LAFEW 6 g	5.96 ± 0.31	5.83 ± 0.30	98.4 ± 4.0
	LAFEW 8 g	6.12 ± 0.47	5.95 ± 0.46	98.1 ± 5.6

Mean ± SE of 5 (Control), 7 (LAFEW 6 g), or 6 (LAFEW 8 g)

Control: Control group, LAFEW 6 g: LAFEW 6 g group, LAFEW 8 g: LAFEW 8 g group

*p < 0.05 vs. 0 week by paired *t*-test, #*p* < 0.05 vs. Control by Dunnett test

*LAFEW* Lactic-fermented egg white

## Discussion

In the present study, 8 weeks of daily consumption of LAFEW with 8 g of EWP was demonstrated to significantly decrease the VF area and visceral fat/subcutaneous fat in subjects with a BMI > 25 compared to the values before consumption or the control group that consumed milk whey protein. When LAFEW was consumed at a daily intake of 6 g as EWP, VF area was not significantly lower than that in the control subjects who consumed whey. A previous double-blinded, placebo-controlled study had reported similar results, indicating that the minimum effective amount of EWP is 8 g/day [6].

In the present study, 8 weeks of consumption of LAFEW was found to decrease the VF area by 13.2 cm^2^. A previous study also revealed an 8.9-cm^2^ decrease in the VF area over the course of a 12-week treatment with daily LAFEW containing 8 g of EWP. Food ingredients reported to reduce visceral fat include catechin [16] and polyphenols [17], and the visceral fat reduction observed under catechin and polyphenol treatments are − 6.5 and 9.4 cm^2^, respectively. Thus, the LAFEW was reported to have a visceral fat-reducing effect equal to or more potent than the functional ingredients known to reduce visceral fat.

In the present study, the visceral fat-reduction effect was observed even in a shorter treatment period compared to that in the previous study. The visceral fat-reduction effect of lactoferrin appears on 8 weeks [8] and the results from the present study suggest that LAFEW has a comparable level of efficacy.

Subjects in this study included few individuals with BMI < 25. When all the subjects were included in the analysis, the LAFEW did not decrease visceral fat compared to the control drink; however, the analysis in subjects with BMI ≥25 revealed a decrease in the VF area, suggesting that LAFEW is more effective in obese individuals than in the healthy individuals. The visceral fat-reducing effect in subjects with BMI > 25 has also been demonstrated in studies of catechin and oolong-tea polyphenols [18, 19]. Apart from visceral fat, the individuals with higher serum levels of total and LDL cholesterol have been reported to have more dramatic results when treated with the functional foods to reduce fat, and the present results are consistent with this finding. Since the metabolic syndrome is often attributed to disturbed eating habits, functional ingredients are presumably less effective for healthy individuals who have regular eating habits and more effective for individuals who have abnormal eating habits [20].

The observed minimum effective amount of EWP consumed to reduce visceral fat was 8 g, suggesting a mechanism closer to that of β-conglycinin, rather than that of lactoferrin. The β-conglycinin is known to suppress lipid absorption, which is the hypothesized mechanism of visceral fat-reduction [9], whereas egg white protein has been similarly reported to suppress lipid absorption. The major components of egg white protein are ovalbumin, ovotransferrin, and lysozyme [10], and its efficacy, albeit in an in vitro study, was comparable. Therefore, the ingredient primarily responsible for suppression of lipid absorption is hypothesized to be ovalbumin, based on its proportion found in egg white. Furthermore, β-oxidation of egg white protein in the liver also increased [4], and we assumed that multiple mechanisms are presumably involved in the visceral fat-reducing effect of LAFEW [4, 9].

In this study, subjects in the control group consumed 8 g of milk whey protein daily, which contains approximately 450 mg of lactoferrin. This amount should be sufficient to reduce visceral fat; however, we did not observe a reduction in visceral fat in the control group. We hypothesize that other proteins in the whey protein may be competing with lactoferrin. Previously, it was reported that consumption of milk whey at even 28 g/day had no effects on visceral fat [21].

The results from the present study involve data for Japanese subjects. Because metabolism differs in diverse genetic backgrounds and with different lifestyles, some optimization of the treatment may be required for it to be successful in diverse populations, including the United States and Europe. Some major cultural differences are potentially lipid intake and BMI. Japanese men with a BMI > 25 are classified as obese, whereas men with BMI > 30 are considered as obese in the United States [22, 23]. The average lipid intake is 62 g among the Japanese men, whereas it is 95 g in the adult males in the United States [24, 25] Considering these two cases, a man in the United States would need to consume an approximate daily intake of LAFEW of about 12 g EWP to reduce visceral fat.

In this study, we also measured the markers of hepatic and renal functions, and found no abnormal increases or decreases. Therefore, these data confirm that consumption at our dosage is safe.

In metabolic syndrome, obesity is thought to trigger pathogenic processes leading to hypertension, dyslipidemia, impaired glucose tolerance, and eventually, arteriosclerotic diseases. If we can proceed with this study and clarify the visceral fat-reducing effect of EWP, the quality of life of obese patients could be improved. We need to do two more experiment to clarify the visceral fat reducing effect of EWP, using subject has different BMI and time dependent change in VS area (as if 2, 4, 6, 8 10,and 12 weeks). Furthermore, if the LAFEW, which is made easier to ingest than original egg white, was commercialized, it could promote public health and reduce visceral fat while allowing for the consumption of a high-quality protein source.

## Conclusion

Minimum effective intake of EWP to reduce the VF area in the Japanese men is 8 g and LAFEW is more effective in obese individuals than healthy individuals.

## Abbreviations

BMI: Body mass index
CT: Computed tomography
EWP: egg white protein
LAFEW: Lactic-fermented egg white
VF: Visceral fat
